# Circularly Polarized Antenna Array with Decoupled Quad Vortex Beams

**DOI:** 10.3390/nano12173083

**Published:** 2022-09-05

**Authors:** Shuo Xu, He-Xiu Xu, Yanzhao Wang, Jian Xu, Chaohui Wang, Zhichao Pang, Huiling Luo

**Affiliations:** Air and Missile Defense College, Air Force Engineering University, Xi’an 710051, China

**Keywords:** antenna array, orbital angular momentum (OAM), vortex beam, multiple beams

## Abstract

Achieving multiple vortex beams with different modes in a planar microstrip array is pivotal, yet still extremely challenging. Here, a hybrid method combining both Pancharatnam−Berry (PB) phase that is induced by the rotation phase and excitation phase of a feeding line has been proposed for decoupling two orthogonal circularly polarized vortex beams. Theoretical analysis is derived for array design to generate quad vortex beams with different directions and an arbitrary number of topological charges. On this basis, two 8 × 8 planar arrays were theoretically designed in an X band, which are with topological charges of *l*_1_ = −1, *l*_2_ = 1, *l*_3_ = −1, and *l*_4_ = 1 in Case I and topological charges of *l*_1_ = −1, *l*_2_ = 1, *l*_3_ = −1, and *l*_4_ = 1 in Case II. To further verify the above theory, the planar array in Case I is fabricated and analyzed experimentally. Dual-LP beams are realized by using rectangular patch elements with two orthogonally distributed feeding networks on different layers based on two types of feeding: proximity coupling and aperture coupling. Both the numerical simulation and experimental measurement results are in good agreement and showcase the corresponding quad-vortex-beam characteristics within 8~12 GHz. The array achieves a measured S_11_ < −10 dB and S_22_ < −10 dB bandwidth of more than 33.4% and 29.2%, respectively. In addition, the isolation between two ports is better than −28 dB. Our strategy provides a promising way to achieve large capacity and high integration, which is of great benefit to wireless and radar communication systems.

## 1. Introduction

Currently, there is an increasing demand on improving the utilization efficiency of spectrum due to the limitation of spectrum and polarization resources. Thus, multiplexing vortex beams carrying orbital angular momentum (OAM) with helical phase have attracted much attention for their infinite number of orthogonal, non-interfering modes at the same frequency [1,2,3,4,5]. It has a wide range of applications in improving the channel capacity of wireless communication systems. The other notable avenue of expanding capacity is integrating multiple functions into a single device [6,7,8,9]. Therefore, multi-mode vortex-beam generation devices would greatly meet the demand of improving spectrum efficiency [10]. Nowadays, various approaches to realize vortex waves have been proposed, such as single microstrip antennas [11,12,13,14,15], annular antenna arrays [16,17,18,19,20], traveling wave antennas [21,22,23,24], and metasurfaces [25,26,27,28,29,30], etc. However, generating multimode vortex beams simultaneously using microstrip array is still in its infancy.

Angular momentum (AM) consists of spin angular momentum (SAM) and OAM, of which SAM is associated with the polarization of electromagnetic waves, the modes of SAM (s = −1, 0, +1) correspond to right-handed circular polarization (RHCP), linear polarization (LP), and left- handed circular polarization (LHCP). The OAM is associated with the spatial phase of helical radio beams. Each helical radio beam has a spatial phase profile with *jlφ*, where *l* is the mode number, also known as topological charge of OAM [31]. In the microstrip array technique, there are two commonly used methods for phase modulation and vortex beam generation: one is using the excitation phase from the feeding network [32,33]; the other is using Pancharatnam−Berry (PB) phase that is generated by element rotation [34,35]. However, both methods only obtain a single mode vortex beam at the same time, and thus there is only one degree of freedom (DoF) for design. Moreover, when the array is synthesized by LP elements, only one circular polarization is operated on the above methods and the other orthogonal polarization is negatively superimposed, so the aperture efficiency limit is only 50%, which may hinder practical applications. Therefore, it is still a difficult challenge to realize vortex beams of different modes in a microstrip array scheme. Fortunately, the PB phase and excitation phase are smartly combined in the generalized sequential rotation microstrip array to produce dual-CP beams by rotating elements and changing the array phase shifter [36], both polarization energies are effectively utilized. However, only dual beams were achieved with one feeding port. Moreover, larger channel capacity, not mention to quad vortex beams that were realized here, cannot be applicable for that scheme because more complicated elements are necessary, which demands stringent solutions for decoupling, and thus would increase the complexity of the integrated system.

Here, we propose an approach to generate quad vortex beams by decoupling dual-LP beams at each feeding port of two. It is composed of two orthogonally fed dual-LP (X and Y polarization) subarrays by combining both the PB phase and excitation phase. Moreover, the appropriate selection of beam pointing and Fourier convolution principle are utilized to independently modulate the four polarized channels (X-LHCP, X-RHCP, Y-LHCP, an Y-RHCP) under two orthogonal LP excitation. For verification, a planar microstrip planar array that is operated at 10 GHz is fabricated, which is capable of emitting quad vortex beams with arbitrary OAM modes of *l*_1_ = −1, *l*_2_ = 1, *l*_3_ = −1, and *l*_4_ = 1. For theoretical design, the approach is further developed for generating decoupled multimode vortex beams which are orthogonal and enable to radiate over a large area. Therefore, the approach will find widespread applications for radars and point-to-point wireless communications and has the potential to be used in communication and imaging systems. The rest of this communication is organized as follows: Section 2 details the decoupled principle for quad vortex beams. In Section 3, the design of the element is presented. Section 4 introduces the feeding line and array design, while the simulated and measured results of the array are given in Section 5. Finally, Section 6 concludes the whole communication.

## 2. Theoretical Background

**Figure 1:**
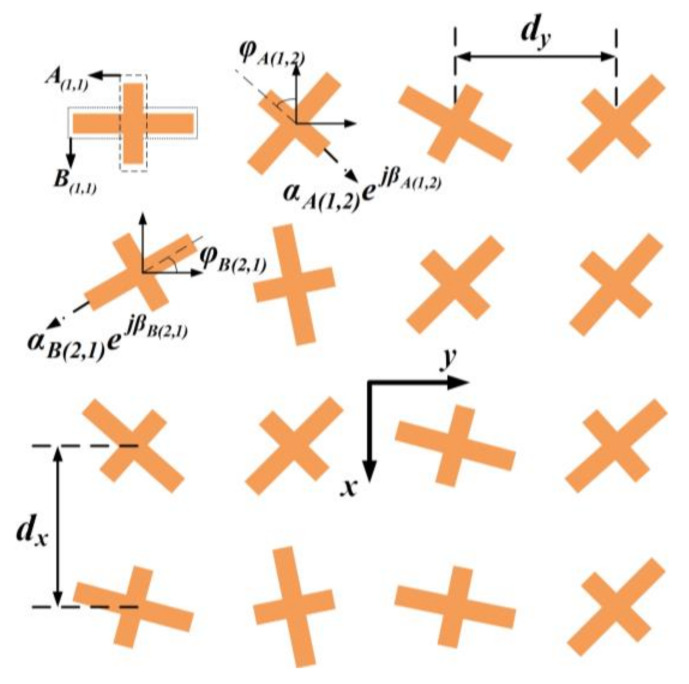
Schematic of planar arrays that are composed of dual LP radiating sub-elements.

In this section, the theory to realize decoupled multi-mode vortex beams is introduced. Figure 1 illustrates the schematic of the proposed dual-LP strategy. The array contains M×N elements with the periods d_x_ and d_y_ along the *x* and *y* axis, respectively. The element can be considered to be composed of two sub-elements that are marked as *A_mn_* and *B_mn_* with different ports, contributing to two orthogonal LP radiations, which can be described as $E_{0,m,n}=\alpha_{m,n}e^{j\beta_{m,n}}\left(e_{L}+e_{R}\right)$, here *m* = 1, 2, …, *M*; *n* = 1, 2, …, *N*. The rotation angle *φ_A(m_*_,*n)*_, *φ_B(m_*_,*n)*_ and excitation phase *β_A(m_*_,*n)*_, *β_B(m_*_,*n)*_ of mn^th^ element are nonuniformly distributed. Here, we take subarray *A* as an example and assume there is no coupling between A and B. The gradients of rotation angles and excitation phases along the *x* and *y* axis are defined as (*φ_x_*, *β_x_*) (*φ_y_*, *β_y_*), and thus the phase distribution can be represented by *φ_A(m_*_,*n)*_ = [*m* − (*M* + 1)/2]*φ_x_* + [*n* − (*N* + 1)/2]*φ_y_* and *β_A(m_*_,*n)*_ = [*m* − (*M* + 1)/2]*β_x_* + [*n* − (*N* + 1)/2]*β_y_*, respectively.

The electric field then turns into the form as [36]:

(1) $$E_{A(m,n)}=R(\varphi_{A(m,n)})E_{0,m,n}\frac{e^{-jkr}}{r}=\alpha_{A(m,n)}(\frac{e^{-j\left(kr-\beta_{A(m,n)}+\varphi_{A(m,n)}\right)}}{r}e_{L}+\frac{e^{-j\left(kr-\beta_{A(m,n)}-\varphi_{A(m,n)}\right)}}{r}e_{R})$$

where $R(\varphi_{A(m,n)})=[\cos\varphi_{A(m,n)},-\sin\varphi_{A(m,n)};\sin\varphi_{A(m,n)},\cos\varphi_{A(m,n)}]$ denotes a rotation factor for coordinate rotation.

In this case, the array electric field is


(2) $$\begin{array}{cc} \; & E_{T}=\frac{\alpha_{0}e^{-jkr}}{r}\left\{\sum_{m=1}^{M}\sum_{n=1}^{N}e^{jk[\left(m-1\right)d_{x}\cos\phi +\left(n-1\right)d_{y}\sin\phi ]\sin\theta }\right\}\left[e^{j(\beta_{A(m,n)}-\varphi_{A(m,n)})}e_{L}+e^{j(\beta_{A(m,n)}+\varphi_{A(m,n)})}e_{R}\right]\\ \; & =\frac{\alpha_{0}e^{j[\left(1-M\right)\left(\beta_{Ax}-\varphi_{Ax}\right)/2+\left(1-N\right)\left(\beta_{Ay}-\varphi_{Ay}\right)/2-kr]}}{r}\left[\sum_{m=1}^{M}e^{j\left(m-1\right)\left(kd_{x}\cos\phi \sin\theta +\beta_{Ax}-\varphi_{Ax}\right)}\sum_{n=1}^{N}e^{j\left(n-1\right)\left(kd_{y}\sin\phi \sin\theta +\beta_{Ay}-\varphi_{Ay}\right)}\right]e_{L}\\ \; & +\frac{\alpha_{0}e^{j[\left(1-M\right)\left(\beta_{Ax}+\varphi_{Ax}\right)/2+\left(1-N\right)\left(\beta_{Ay}+\varphi_{Ay}\right)/2-kr]}}{r}\left[\sum_{m=1}^{M}e^{j\left(m-1\right)\left(kd_{x}\cos\phi \sin\theta +\beta_{Ax}+\varphi_{Ax}\right)}\sum_{n=1}^{N}e^{j\left(n-1\right)\left(kd_{y}\sin\phi \sin\theta +\beta_{Ay}+\varphi_{Ay}\right)}\right]e_{R} \end{array}$$

In this regard, the LHCP wave and RHCP wave with different array factors (*AF_L_*, *AF_R_*) will be generated from the decoupled LP wave, and *AF_L_* and *AF_R_* are formulated respectively as:

(3) $$\begin{array}{l} AF_{L}=De^{j(\beta_{A(m,n)}-\varphi_{A(m,n)})}\\ =\sum_{m=1}^{M}e^{j(m-1)(kd_{x}\cos\phi_{L}\sin\theta_{L}+\beta_{x}-\varphi_{x})}\sum_{n=1}^{N}e^{j(n-1)(kd_{y}\sin\phi_{L}\sin\theta_{L}+\beta_{y}-\varphi_{y})} \end{array}$$

(4) $$\begin{array}{l} AF_{R}=De^{j(\beta_{A(m,n)}+\varphi_{A(m,n)})}\\ =\sum_{m=1}^{M}e^{j(m-1)(kd_{x}\cos\phi_{R}\sin\theta_{R}+\beta_{x}+\varphi_{x})}\sum_{n=1}^{N}e^{j(n-1)(kd_{y}\sin\phi_{R}\sin\theta_{R}+\beta_{y}+\varphi_{y})} \end{array}$$

where $D=\sum_{m=1}^{M}\sum_{n=1}^{N}e^{jk[(m-1)d_{x}\cos\phi +(n-1)d_{y}\sin\phi ]\sin\theta }$, (*θ*_L_,*ϕ*_L_), and (*θ*_R_, *ϕ*_R_) are azimuthal and elevation angles of LHCP and RHCP beams in free space. To form an efficient beam, the intensity of *AF_L_* and *AF_R_* should be a real number which requires that the combined phase term in each array factor is zero. Therein, the gradients of rotation angle and excitation phase between adjacent elements are derived as a function of the azimuthal and elevation angles.

(5) $$\varphi_{x}=kd_{x}\left(\sin\theta_{L}\cos\phi_{L}-\sin\theta_{R}\cos\phi_{R}\right)/2\;\beta_{x}=kd_{x}\left(-\sin\theta_{L}\cos\phi_{L}-\sin\theta_{R}\cos\phi_{R}\right)/2\;\varphi_{y}=kd_{y}\left(\sin\theta_{L}\sin\phi_{L}-\sin\theta_{R}\sin\phi_{R}\right)/2\;\beta_{y}=kd_{y}\left(-\sin\theta_{L}\cos\phi_{L}-\sin\theta_{R}\sin\phi_{R}\right)/2$$

It can be seen from above derivation that the LP wave can be decoupled into dual-CP beams by changing the rotation angle of elements, which is the same as the PB phase. And the combination of rotation angle and excitation phase can control the beam directions.

To implement radio beams carrying OAM, the planar array should exhibit a spiral phase pattern *e^jl^^ϕ^*, where *ϕ* = $\;\tan^{-1}\frac{y}{x}$ is the phase-shift factor, *ϕ* is the azimuthal angle around *z* axis, and (*x*, *y*) are the positions of element. When a linear gradient phase pattern is superimposed with a spiral phase profile, a tilted vortex beam will form and the deflection angle will be determined by the linear gradient phase [37]. Similarly, when a vortex phase profile is superimposed to the linear excitation phase, the deflected vortex beam can be formed, i.e., $\beta_{A\left(m,n\right)}^{\prime }=l_{1}\tan^{-1}(\frac{y}{x})+\beta_{A\left(m,n\right)}$. In that case, the array factors of dual-CP can be expressed as:

(6) $$AF_{L}{}^{\prime }=De^{j[\beta_{A(m,n)}+l_{1}\tan^{-1}(\frac{y}{x})-\phi_{A(m,n)}]}=AF_{L}e^{j[l_{1}\tan^{-1}(\frac{y}{x})]}\;AF_{R}{}^{\prime }=De^{j[\beta_{A(m,n)}+l_{1}\tan^{-1}(\frac{y}{x})+\phi_{A(m,n)}]}=AF_{R}e^{j[l_{1}\tan^{-1}(\frac{y}{x})]}$$

Moreover, the above vortex phase can also be superimposed with the linear PB phase, i.e., $\varphi_{A\left(m,n\right)}^{\prime }=l_{2}\tan^{-1}(\frac{y}{x})+\varphi_{A\left(m,n\right)}$. Then, the array factors of dual-CP wave can be formulated as:

(7) $$AF_{L}{}^{\prime \prime }=De^{j[\beta_{A(m,n)}-\phi_{A(m,n)}-l_{1}\tan^{-1}(\frac{y}{x})]}=AF_{L}e^{j[-l_{2}\tan^{-1}(\frac{y}{x})]}\;AF_{R}{}^{\prime \prime }=De^{j[\beta_{A(m,n)}+\phi_{A(m,n)}+l_{1}\tan^{-1}(\frac{y}{x})]}=AF_{R}e^{j[l_{2}\tan^{-1}(\frac{y}{x})]}$$

According to above theoretical analysis, dual circularly polarized vortex beams with identical topological charges will be generated in the total radiation field when a spiral phase is imposed to the excitation phase. However, dual-CP vortex beams will be generated with opposite topological charges when the spiral phase pattern is imposed to the rotation angle. Equations (6) and (7) predict that decoupled dual vortex beams can be generated independently in both subarrays A and B. By simultaneously exciting both arrays, quad vortex beams can be engineered with different directions.

## 3. Dual-LP Element Design

**Figure 2:**
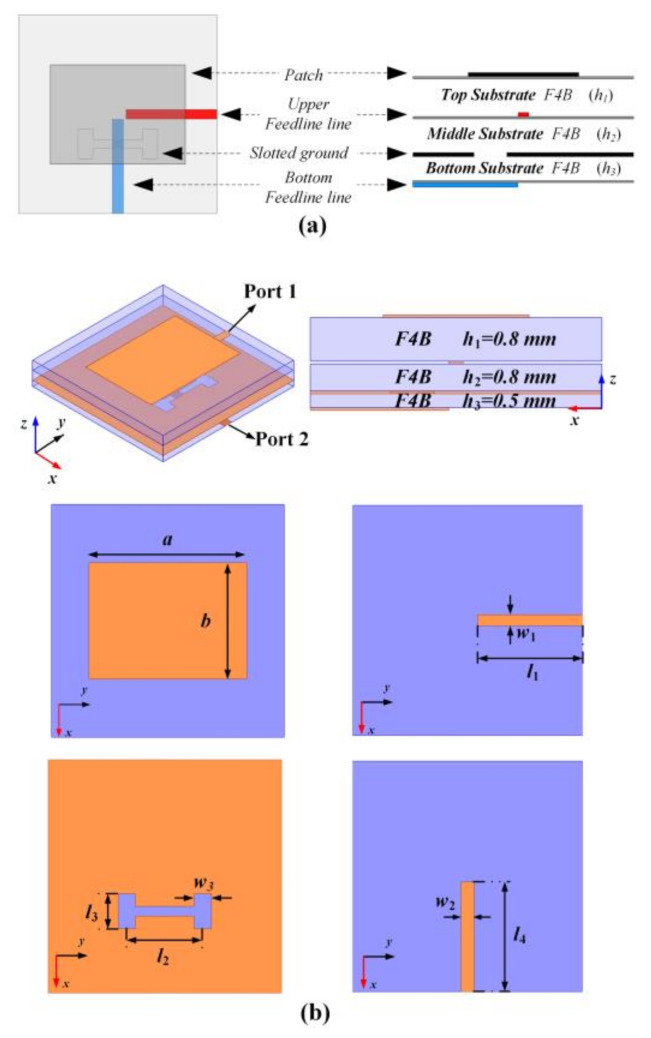
Geometry of the proposed dual-LP element. (**a**) Schematic diagram. (**b**) Layout and parametric illustration. The optimized dimensions are: *a* = 8.15 mm, *b* = 6 mm, *w*_1_ = 0.56 mm, *w*_2_ = 0.67 mm, *l*_1_ = 5.5 mm, *l*_2_ = 3.9 mm, *l*_3_ = 1.8 mm, *l*_4_ = 5.7 mm, *h*_1_ = 0.8 mm, *h*_2_ = 0.8 mm, and *h*_3_ = 0.5 mm.

To independently generate dual orthogonal LP radiations, a dual-feed element is presented, see Figure 2. The element is composed of four conductive layers that are separated by three F4B dielectric substrates (ε_r_ = 2.65 + *j*0.001). As shown in Figure 2a, the dual-feed element can be decomposed as two sub-elements sharing an identical rectangular patch and ground plane with an H-shaped slot. To increase the isolation between the two sub-elements and avoid the crosstalk of the feeding networks, two ports are orthogonally arranged and printed in different layers to transmit dual LP wave radiations with orthogonal polarization. The Y-polarization operation of sub-element A is realized by proximity feeding using the upper feeding line, while the X-polarized operation of sub-element B is realized by aperture coupling using the bottom feeding line. The layout and parametric illustration are given in Figure 2b.

**Figure 3:**
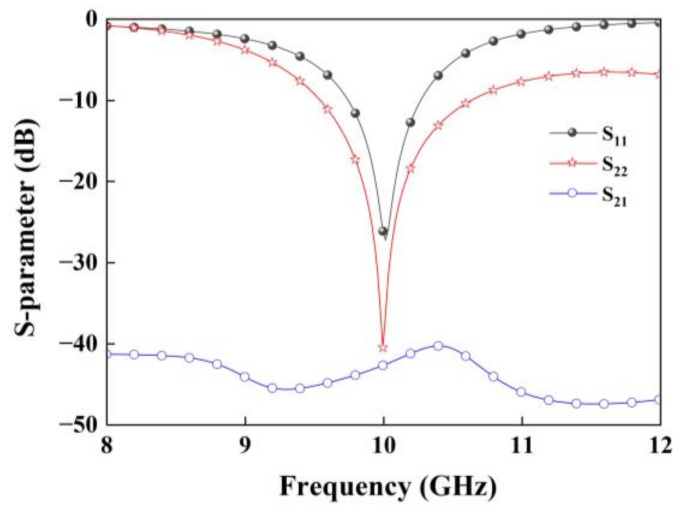
Numerically simulated S-parameters of the dual-LP patch element.

The element is numerically simulated by the simulation package of High Frequency Structure Simulator (HFSS), and the simulated S-parameters of the proposed dual-LP element is shown in Figure 3. In calculations of S parameter, we only calculate an element with radiation boundary conditions that are imposed at distance 10 mm away from the element, while two lumped ports placed at Port 1 and Port 2 to transmit orthogonal linearly polarized beams. It can be seen that the simulated |S_11_| and |S_22_| below −10 dB are observed over the bandwidths of 9.81–10.35 GHz and 9.56–10.67 GHz, and the isolation between the two ports is better than −38 dB.

**Figure 4:**
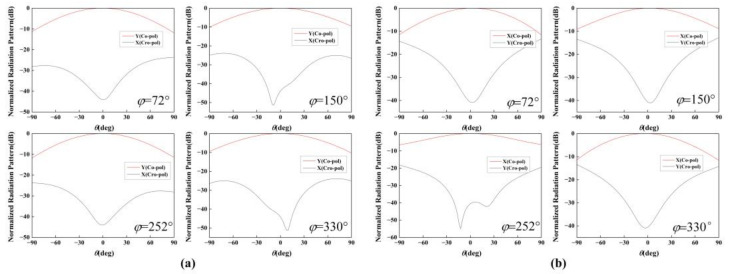
Normalized co-polarization and the cross-polarization patterns for LP element when the (**a**) sub-element A and (**b**) sub-element B working in *ϕ* = 72°, 150°, 252°, 330°.

The co-polarization and cross-polarization radiation patterns of the sub-element that is fed separately by Port 1 and Port 2 in cartesian coordinate system are illustrated at 10 GHz in Figure 4. For convenience and clear identification, all far-field patterns at each deflection angle were normalized to their maximal intensity of co-polarized components at an azimuthal plane of *ϕ* = 72°, 150°, 252°, and 330°, where four vortex beams will be deposited. As plotted in Figure 4a, when Port 1 is fed separately, the co-polarization component (Y) remains above −3 dB in range ±44°, and in this region, the intensity of the cross-polarization component (X) is below −27 dB in the plane of *ϕ* = 72°, 150°, 252°, and 330°. In contrast, the co-polarization component (X) remains above −3 dB in the range of ±42° when Port 2 is fed separately, and in this region the cross-polarization component(Y) intensity is below −23 dB in the plane of *ϕ* = 72°, 150°, 252°, and 330°, indicating that the element that is fed by both two ports exhibits high LP performance.

## 4. Design of Quad-Vortex-Beam Planar Array

**Figure 5:**
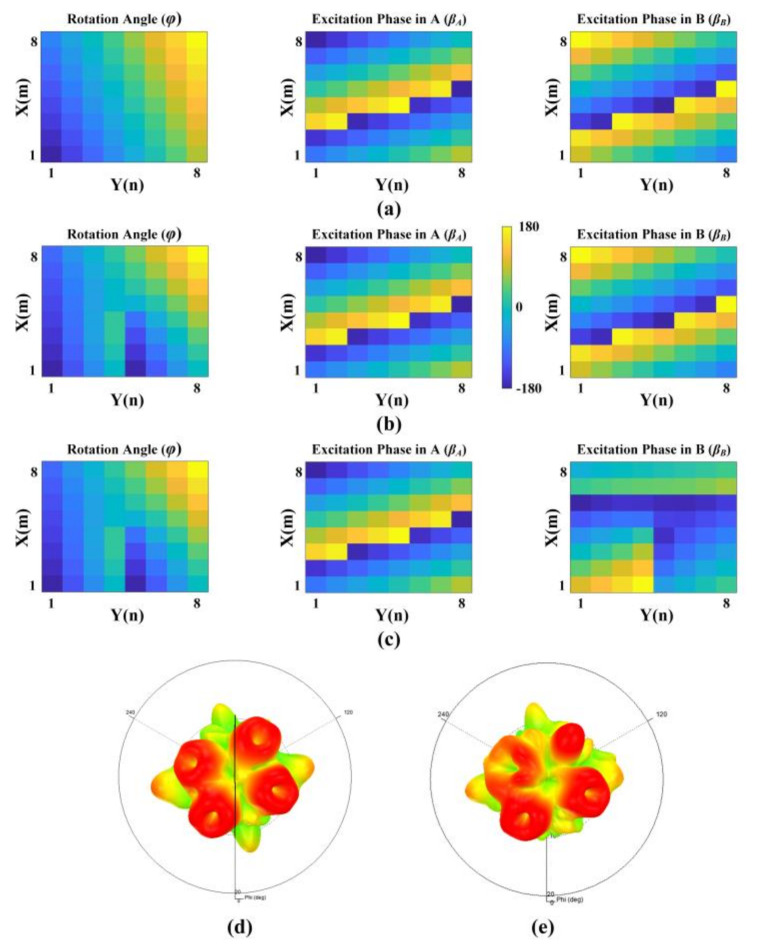
Rotation angles and excitation phases of sub-elements A and B for (**a**) quad CP beams without OAM and (**b**,**c**) quad-vortex-beam with (**b**) *l_(A_*_,_ *_L)_*= *l_(B_*_,_ *_L)_*= −1, *l_(A_*_,_ *_R)_*= *l_(B_*_,_ *_R)_*= 1 in Case I and (**c**) *l_(A_*_,_ *_L)_*= −1, *l_(B_*_,_ *_L)_*= 0, *l_(A_*_,_ *_R)_*= 2, *l_(B_*_,_ *_R)_*= 1. in case II (**d**,**e**) Simulated far-field patterns (**d**) in Case I and (**e**) in case II.

In this section, the planar microstrip quad-vortex-beam patch arrays are designed at 10 GHz. According to Equation (3), the directions of the vortex beams can be realized by combining rotation angle and excitation phase. Here, specifically two corresponding sub-elements of array A and array B are designed to exhibit the same rotation angle for easy design. To reduce mutual coupling and the interference among the resulting beams, it is necessary to select appropriate numerical solutions of Equation (3) to set four vortex beams in different quadrants. The deflection angles of LHCP/RHCP beams that are generated by subarray A and B are designed at *θ*_(*A*,_ *_L_*_)_ = 30°, *ϕ*_(*A*,_ *_L_*_)_ = 72°; *θ*_(*B*,_ *_L_*_)_ = 30°, *ϕ*_(*B*,_ *_L_*_)_ = 150°; *θ*_(*B*,_ *_R_*_)_ = 30°, *ϕ*_(*B*,_ *_R_*_)_ = 252°; *θ*_(*A*,_ *_R_*_)_ = 30°, and *ϕ*_(*A*,_ *_R_*_)_ = 330°. In this case, the distributions of the rotation angle and excitation phase are shown in Figure 5a.

The element that is presented in Section 3 contains three DoFs, namely the rotation angle (*φ*), and two excitation phases (*β_A_*, *β_B_*) that are introduced by feeding lines of two different sub-elements. According to Equations (4) and (5), the final quad-vortex-beam will obtain different topological charges (*l_(A_*_,_ *_L)_*, *l_(A_*_,_ *_R)_*, *l_(B_*_,_ *_L)_*, *l_(B_*_,_ *_R)_*) when the vortex phase profile with topological charges of *l*_1_, *l*_2_, and *l*_3_ is superimposed to the above three phase patterns, respectively. Table 1 summarizes all of the scenarios of topological charges of the final vortex beams relative to three DoFs.

As a proof, we demonstrate two cases in Table 1 to achieve a quad-vortex-beam at 10 GHz. In both cases, the arrays are composed of 8 × 8 elements with a period of *d_x_* = *d_y_* = 18 mm. The distributions of the rotation angles and excitation phases are illustrated in Figure 5b,c. For Case I, *l_1_* = 1, *l_2_* = *l_3_* = 0 and then *l_(A_*_,_ *_L)_*= *l_(B_*_,_ *_L)_*= −1, *l_(A_*_,_ *_R)_*= *l_(B_*_,_ *_R)_*= 1 is obtained according to Table 1; for Case II, *l_1_* = 1, *l_2_* = 0, *l_3_* = 1 is chosen, and thus *l_(A_*_,_ *_L)_*= −1, *l_(B_*_,_ *_L)_*= 0, *l_(A_*_,_ *_R)_*= 2, *l_(B_*_,_ *_R)_*= 1. Figure 5d,e plot the corresponding far-field patterns that were simulated in HFSS, where the patterns are obtained by an API process which can automatically construct all the metallic patterns through program codes introducing 128 excitation phases to 128 ports with specific rotation angles, rather than using real feeding lines.

**Figure 6:**
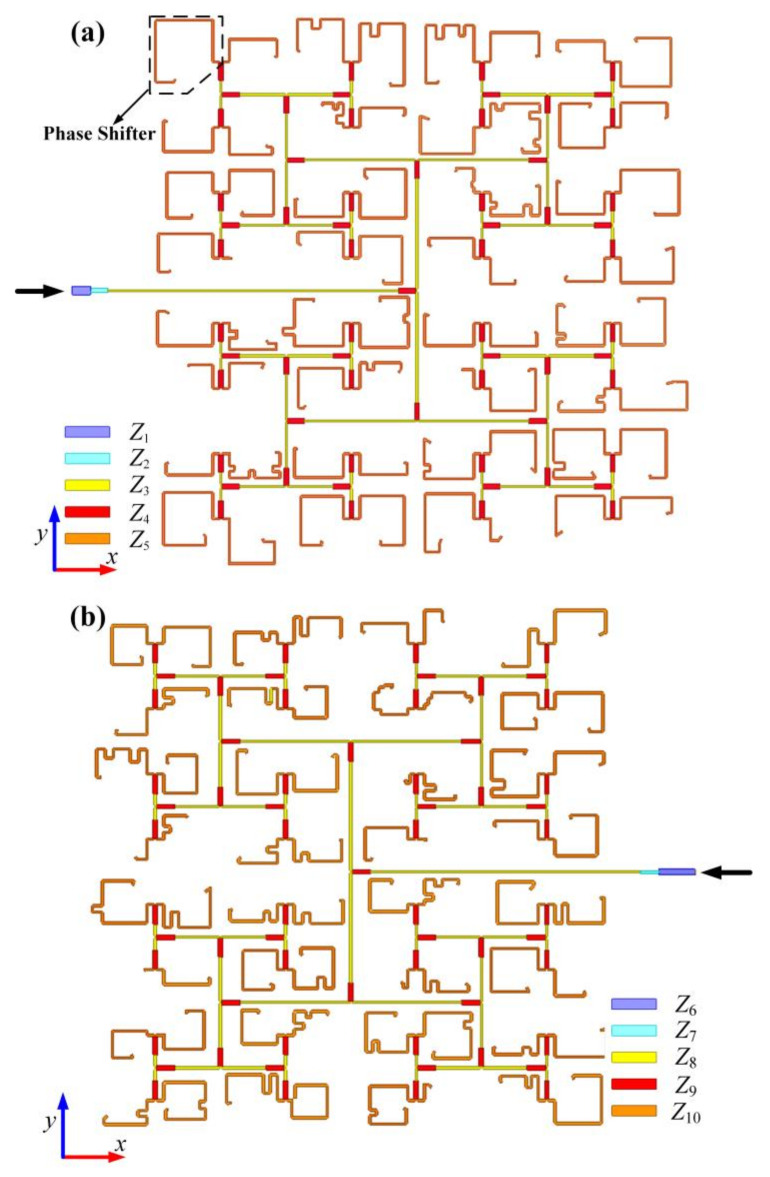
Layouts of the (**a**) upper and (**b**) bottom feeding network for the quad-vortex-beam array in Case I.

**Figure 7:**
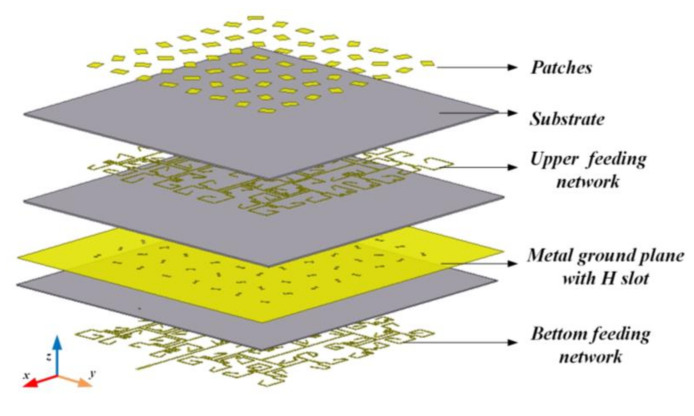
3-D view of the planar microstrip quad-vortex patch array in Case I.

To further illustrate the effectiveness of the approach in generating a decoupled quad-vortex-beam, we conduct a complete full-wave simulation design for Case I using real feeding lines. To realize the above excitation phase, two sets of feeding networks are designed as shown in the Figure 6, which are composed of six-order power dividers based on T-junction structures, and 64 phase shifters that are realized by microstrip lines with different lengths. The feeding lines with different characteristic impedances (*Z*) are denoted by different colors, and the corresponding widths are shown in Table 2 and Table 3. Since all the elements exhibit different rotation angles and different excitation phases, all the feeding lines should be carefully designed to fulfil the theoretical excitation phases and the requirement of rotations. It should be noted that the total electric field remains unchanged according to Equation (2) when both the excitation phase and the rotation angle change by 180°. Therefore, some elements can be rotated 180° to increase or decrease the excitation phase by 180° if the excitation phase of elements is difficult to meet the requirements in the finite circuit area. Figure 7 shows the final layout of the planar microstrip quad-vortex-beam patch array. The ports of subarray A and subarray B are located at both sides of the *yoz* plane, and the ground plane is extended out slightly from the edge elements to solder SMA connectors. As it can be seen, the patches and feeding lines varied point by point.

**Figure 8:**
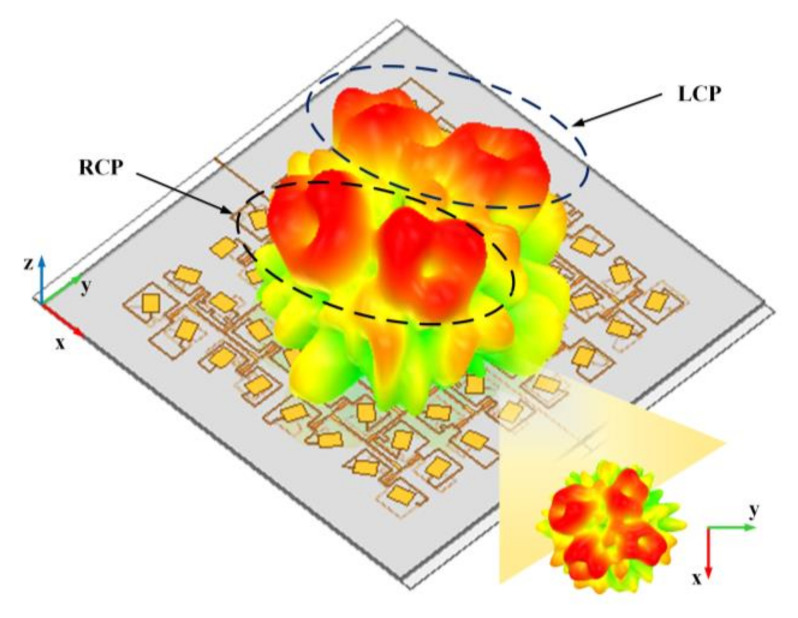
Simulated 3-D radiation pattern at 10 GHz for the quad-vortex-beam in Case I with different OAM modes (*l_(A_*_,_ *_L)_*= *l_(B_*_,_ *_L)_*= −1, *l_(A_*_,_ *_R)_*= *l_(B_*_,_ *_R)_*= 1) at predicted deflection angles of (*θ*, *ϕ*) = (30°, 72°), (30°, 150°), (30°, 252°), and (30°, 330°).

For demonstration, the simulated 3-D far-field pattern when the two ports are fed simultaneously at 10 GHz is depicted in Figure 8. There are four doughnut patterns that are clearly observed with identical aperture size, indicating four effectively formed vortex beams. There are two LHCP vortex beams that are generated from subarray A and subarray B that are located at *θ_(A_*_,_ *_L)_*= 30°, *ϕ_(A_*_,_ *_L)_*= 72°, and *θ_(B_*_,_ *_L)_*= 30°, *ϕ_(B_*_,_ *_L)_*= 150° with topological charges of *l_(A_*_,_ *_L)_*= *l_(B_*_,_ *_L)_*= −1, while two RCP vortex beams are located at *θ_(B_*_,_ *_R)_*= 30°, *ϕ_(B_*_,_ *_R)_*= 252°; *θ_(A_*_,_ *_R)_*= 30°, and *ϕ_(A_*_,_ *_R)_*= 330° with topological charges of *l_(A_*_,_ *_R)_*= *l_(B_*_,_ *_R)_*= 1.

The co-polarization and cross-polarization radiation patterns of the sub-element that was fed separately by Port 1 and Port 2 in cartesian coordinate system are illustrated at 10 GHz in Figure 4. For convenience and clear identification, all the far-field patterns at each deflection angle were normalized to their maximal intensity of co-polarized components at an azimuthal plane of *ϕ* = 72°, 150°, 252°, and 330°, where four vortex beams will be deposited. As plotted in Figure 4a, when Port 1 is fed separately, the co-polarization component (X) remains above −3 dB in range ±44°, and in this region, the intensity of the cross-polarization component (Y) is below −27 dB in the plane of *ϕ* = 72°, 150°, 252°, and 330°. In contrast, the co-polarization component (X) remains above −3 dB in the range of ±42° when Port 2 is fed separately, and in this region the cross-polarization component (Y) intensity is below −23 dB in the plane of *ϕ* = 72°, 150°, 252°, and 330°, indicating that the element that is fed by both two ports exhibits high LP performance.

## 5. Results and Discussion

**Figure 9:**
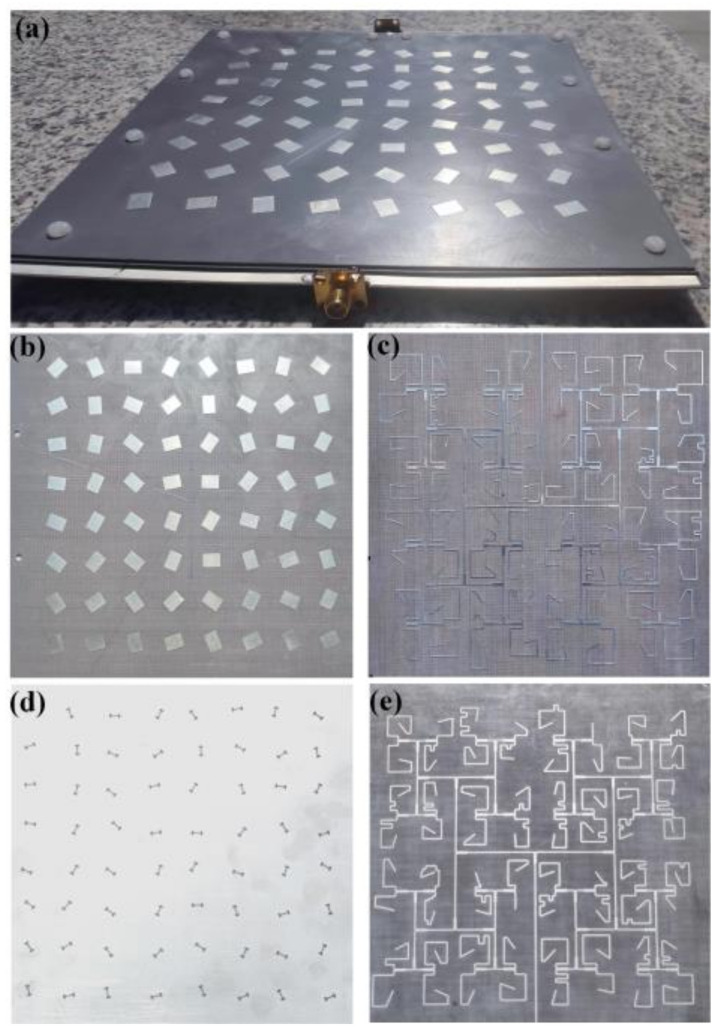
Photographs of the final fabricated quad-vortex-beam planar microstrip array. (**a**) Assembled model, (**b**) radiating elements, (**c**) upper feeding network, (**d**) ground plane with H slot, and (**e**) bottom feeding network.

**Figure 10:**
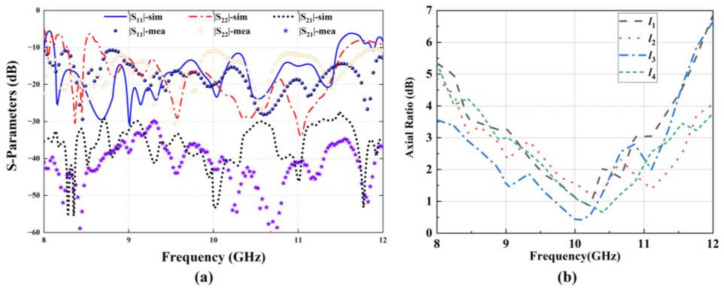
(**a**) Simulated and measured S-parameters and (**b**) simulated AR of the microstrip quad-vortex-beam planar array.

To validate the proposed design concept, the quad-vortex-beam planar array is fabricated using the printed-circuit-board (PCB) technology, as shown in Figure 9a. The prototype is assembled by nylon screws with the overall size of 180 mm × 190 mm. Figure 9b–e shows the photographs of the array for each layer. Figure 10a presents the simulated and measured impedance matching performance and the transmission response between the two ports. It shows that the impedance bandwidth of S_11_ ≤ −10 dB is 3.34 GHz (8.12–11.46 GHz), with a relative bandwidth of 33.4%; the impedance bandwidth of S_22_ ≤ −10 dB is 2.92 GHz (8.66–11.58 GHz), corresponding to a relative bandwidth of 29.2%. The transmission coefficient of S_21_ is lower than −28 dB over 8–12 GHz, indicating an elegant isolation. Slight differences of results between simulations and measurements are due to small gaps between dielectric substrates and some errors of relative positions of the dielectric substrates when they are assembled. Figure 10b shows the simulated axial ratios of *l*_1_ = −1 (Theta = 21°, Phi = 72°), *l*_2_ = 1 (Theta = 20°, Phi = 150°), *l*_3_ = −1 (Theta = 20°, Phi = 252°), and *l*_4_ = 1 (Theta = 22°, Phi = 330°). The simulated axial ratio (AR) bandwidth of quad modes is about 17%.

**Figure 11:**
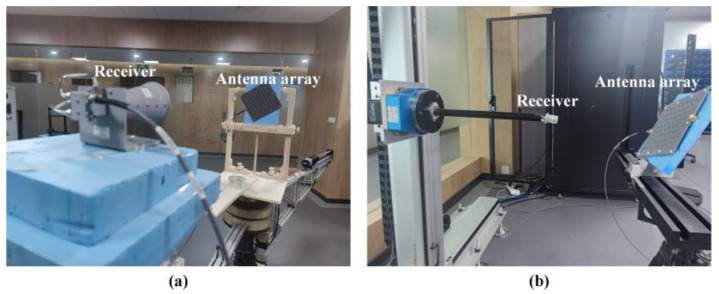
Experimental setup for (**a**) far-field and (**b**) near-field measurement.

Figure 11 shows the far-field and near-field measurement setups. All the experiments are performed in free space. In the far-field measurement, a dual CP horn with an axial ratio less than 3.5 dB over 6–18 GHz and VSWR ≤ 2.5 is utilized as a receiver. The planar microstrip array is set at different azimuthal planes of *ϕ* = 72°, 150°, 252°, and 330° in front of the receiver, and both are connected to an AV3672B vector network analyzer to generate and receive signals. In the near-field experiment, a planar spiral antenna is displaced with a distance of 0.25 m from the central axis of the sample, and functions as the receiver and moves automatically in a maximum area of 0.2 m × 0.2 m. The microstrip quad-vortex-beam planar array is first rotated along the *z* axis by *ϕ* and then along the *y* axis by *θ* to align the central axis of the vortex beam with the receiver.

**Figure 12:**
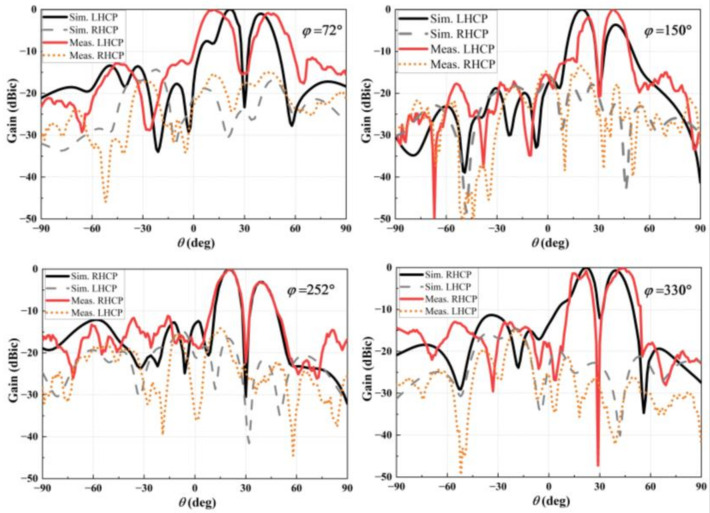
Numerical far-field patterns of the LHCP and RHCP beams in the *ϕ*_(*A*,_ *_L_*_)_ = 72°; *ϕ*_(*B*,_ *_L_*_)_ = 150°; *ϕ*_(*B*,_ *_R_*_)_ = 252°; and *ϕ*_(*A*,_ *_R_*_)_ = 330° planes at 10 GHz.

Figure 12 plots the simulated and measured normalized far-field radiation patterns at 10 GHz, where both results agree well. It can be seen that the LCP beam is excited in directions of *ϕ*_(*A*,_ *_L_*_)_ = 72°, *θ*_(*A*,_ *_L_*_)_ = 30°, and *ϕ*_(*B*,_ *_L_*_)_ = 150°, *θ*_(*B*,_ *_L_*_)_ = 30°. In contrast, the RCP wave is formed in directions of *ϕ*_(*B*,_ *_R_*_)_ = 252°, *θ*_(*B*,_ *_R_*_)_ = 30°, and *ϕ*_(*A*,_ *_R_*_)_ = 330°, *θ*_(*A*,_ *_R_*_)_ = 30°. In each beam, there is an intensity null at the corresponding main lobe. The sidelobe levels are observed as −12 dB, −14 dB, −15 dB, and −18 dB for the co-polarization component in an azimuthal angle of *ϕ*_(*A*,_ *_L_*_)_ = 72°, *ϕ*_(*B*,_ *_L_*_)_ = 150°, *ϕ*_(*B*,_ *_R_*_)_ = 252°, and *ϕ*_(*A*,_ *_R_*_)_ = 330°, respectively.

**Figure 13:**
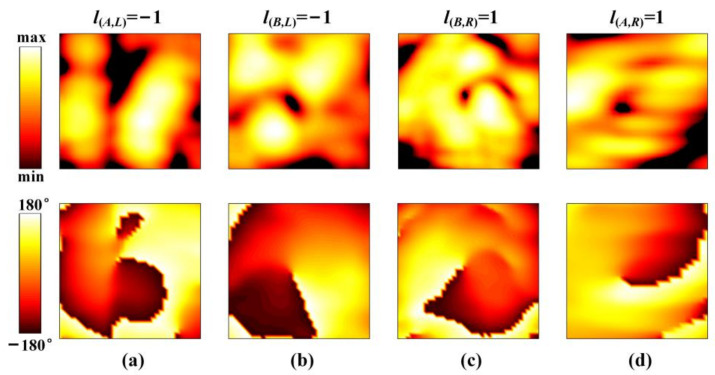
The measured magnitude and phase distributions at 10 GHz for four beams of different OAM modes at different directions. (**a**) *l*_(*A*,_ *_L_*_)_ = 1 at *θ*_(*A*,_ *_L_*_)_ = 30°, *ϕ*_(*A*,_ *_L_*_)_ = 72°. (**b**) *l*_(*B*,_ *_L_*_)_ = 1 at *θ*_(*B*,_ *_L_*_)_ = 30°, *ϕ*_(*B*,_ *_L_*_)_ = 150°. (**c**) *l*_(*B*,_ *_R_*_)_ = −1 at *θ*_(*B*,_ *_R_*_)_ = 30°, *ϕ*_(*B*,_ *_R_*_)_ = 252°. (**d**) *l*_(*A*,_ *_R_*_)_ = −1 at *θ*_(*A*,_ *_R_*_)_ = 30°, *ϕ*_(*A*,_ *_R_*_)_ = 330°.

Figure 13 shows the measured near-field magnitude and phase maps at 10 GHz. The intensity null in the center of the beams is evident, which shows the main characteristic of the OAM vortex beam. Moreover, the phase changes counterclockwise from 180° to −180° in Figure 13a,b, which corresponds to the feature of *l*_(*A*,_ *_L_*_)_ = −1, *l*_(*B*,_ *_L_*_)_ = −1, while it varies clockwise from 180° to −180° in Figure 13c,d corresponding to *l*_(*A*,_ *_R_*_)_ = *l*_(*B*,_ *_R_*_)_ = 1. Moreover, the spatial phase fronts are slightly distorted with an irregular doughnut shape, which is caused by the deflection and the side-lobe interference of other beams. Nevertheless, all the results have verified our proposed method in generating decoupled quad vortex beams with topological charges of *l*_(*A*,_ *_L_*_)_ = *l*_(*B*,_ *_L_*_)_ = −1, *l*_(*A*,_ *_R_*_)_ = *l*_(*B*,_ *_R_*_)_ = 1.

## 6. Conclusions

To sum up, we have proposed and demonstrated a strategy in generating quad decoupled vortex beams by using a CP antenna array. The relationship among the OAM modes, rotation angle, and the excitation phase of the array elements is analyzed. For a demonstration, a microstrip antenna array with topological charges of *l*_(*A*,_ *_L_*_)_ = *l*_(*B*,_ *_L_*_)_ = −1, *l*_(*A*,_ *_R_*_)_ = *l*_(*B*,_ *_R_*_)_ = 1 at 10 GHz is fabricated and measured. All the results agree well and show that the proposed strategy was capable of realizing multi-mode decoupled vortex beams in broadband. The proposed device features compactness, wide bandwidth, and large channel capacity, promising potential engineering applications in radars and point-to-point wireless communications.

## Figures and Tables

**Table 1:** Relationship of Topological Charges of the Final Vortex Beams to Three DoFs.

	*l*_(*A*,_ *_L_*_)_	*l*_(*A*,_ *_R_*_)_	*l*_(*B*,_ *_L_*_)_	*l*_(*B*,_ *_R_*_)_
*l*_1_*ϕ* in *φ*	−*l*_1_	*l*_1_	*−l*_1_	*l*_1_
*l*_2_*ϕ* in *β_A_*	*l*_2_	*l*_2_	/	/
*l*_3_*ϕ* in *β_B_*	/	/	*l*_3_	*l*_3_
Final topological charge	−*l*_1_ + *l*_2_	*l*_1_ + *l*_2_	*l*_1_ + *l*_3_	*l*_1_ + *l*_3_

**Table 2:** Parameters of the upper feeding network.

	*Z*_1_	*Z*_2_	*Z*_3_	*Z*_4_	*Z*_5_
*Z* (Ω)	50	67	92	65	92
Width (mm)	2.25	1.25	0.56	1.3	0.56
Length (mm)	5	4.7	3.97~80.27	4.75	/

**Table 3:** Parameters of the bottom feeding network.

	*Z*_6_	*Z*_7_	*Z*_8_	*Z*_9_	*Z*_10_
*Z* (Ω)	50	61	75	53	75
Width (mm)	1.43	0.86	0.67	1.23	0.67
Length (mm)	10	5.08	3.6~74.5	5.06	/

## Data Availability

The data that support the findings of this study are available from the corresponding author upon reasonable request.
